# A Microscopy Study of the Structural Features of Post-LASIK Human Corneas

**DOI:** 10.1371/journal.pone.0063268

**Published:** 2013-05-01

**Authors:** Mohammad Abahussin, Sally Hayes, Henry Edelhauser, Daniel G. Dawson, Keith M. Meek

**Affiliations:** 1 Structural Biophysics Research Group, School of Optometry and Vision Sciences, Cardiff University, Cardiff, United Kingdom; 2 Optometry Department, College of Applied Medical Science, King Saud University, Riyadh, Saudi Arabia; 3 Emory Eye Center, Emory University, Atlanta, Georgia, United States of America; 4 Department of Ophthalmology, University of Florida, Gainsville, Florida, United States of America; University of Missouri-Columbia, United States of America

## Abstract

**Purpose:**

To study the structural features of human post-LASIK corneas.

**Methods:**

A pair of post-mortem donor corneas, from a 55-year old patient who underwent uncomplicated LASIK surgery five years previously, were bisected and fixed in 4% paraformaldehyde. The right cornea and one half of the left cornea were processed for light microscopy and scanning electron microscopy. One half of the right cornea was also examined by transmission electron microscopy.

**Results:**

The flap-bed interface could be easily detected several years after LASIK and, although the flap appeared to be in close association with the stromal bed, there was a noticeable absence of reconnection between adjacent severed lamellae. Tissue gaps were evident at the flap margin, which once free of cellular components revealed the presence of a few bridging fibres.

**Conclusion:**

Examination of corneas five years after LASIK revealed evidence of primitive reparative scar development at the wound interface, but no reconnection of severed collagen lamellae. Such findings may explain the occurrence of flap dislocation following trauma in some patients months or years after surgery.

## Introduction

**Figure 1:**
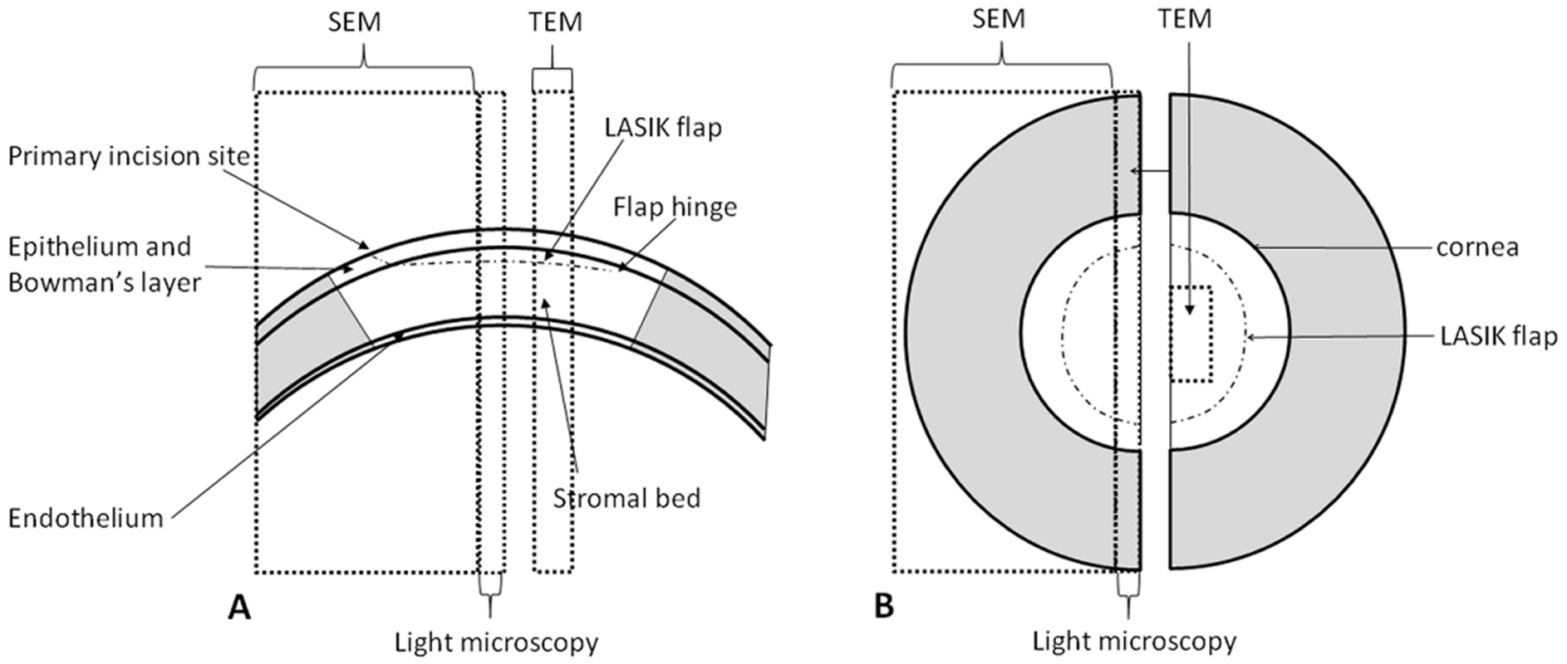
Schematic cross-sectional (A) and overhead (B) view of the bisected right post-LASIK corneo-scleral button. Regions examined by scanning electron microscopy (SEM), transmission electron microscopy (TEM) and light microscopy have been highlighted.

Laser in situ keratomileusis (LASIK) has been used to correct corneal refractive error for over 20 years [1]; the surgery involves the use of a mikrokeratome to create a hinged corneal flap and the *in situ* ablation of the exposed stromal bed with an excimer laser (Figure 1A). In the literature, a large number of publications have evaluated the results of LASIK surgery in terms of its clinical outcomes. However, few have investigated the ultrastructural changes that occur within the cornea and result in the creation of a flap that can be easily dislocated many years after treatment [2], [3]. Moreover, most studies that concentrate on ultrastructural changes are based on animal models [4], [5], [6] and one must realise that the architecture [7] and material properties [8] of animal corneas can be quite different from those of humans.

This paper examines the corneal ultrastructure of two post-mortem human corneas that underwent uncomplicated LASIK 5 years previously. The findings contribute to our understanding of the material behavior of post-LASIK corneas.

## Methods

### Ethics statement

The research presented in this manuscript was approved by the Human Science Ethical Committee (School of Optometry and Vision Sciences, Cardiff University, UK). The institutional review board approved the use of all corneas described in this study; a waiver of consent was given for the donor corneas as they were obtained from an eye bank (Georgia Eye Bank, Atlanta, USA). All tissue used in this study was obtained in accordance with the tenets of the Declaration of Helsinki and local ethical rules were adhered to throughout.

### Tissue

Two healthy corneo-scleral buttons from a 55 year old male donor who underwent uncomplicated bilateral LASIK surgery five years prior to death were provided by the Georgia Eye Bank for the purposes of this study. No other ocular history details, such as refractive errors and corneal topography maps, were available from the eye bank. The corneo-scleral buttons were stored for 7 days in Optisol-GS solution (Bausch and Lomb Rochester, NY) before being fixed in 4% paraformaldehyde for one week. The corneo-sclera buttons were then bisected and half of the left cornea and both halves of the right cornea sent to the UK. On arrival, the samples were stored for a further two weeks in 4% paraformaldehyde before being processed for light microscopy and scanning electron microscopy. The right cornea was also processed for transmission electron microscopy.

### Light Microscopy

A 2 mm limbus to limbus section was cut from each cornea (Figure 1) and embedded in wax. Thin corneal cross sections (7 µm thick) were cut from the wax blocks and mounted on slides. The corneal sections were wax cleared and dehydrated before being stained with Haematoxylin and Eosin and examined under a light microscope (DMRAZ, Leica Milton, UK).

### Scanning electron microscopy

One half of each corneo-sclera button (Figure 1) was immersed in 2.5% glutaraldehyde for 2 hours. The tissues were then placed into a solution of 5% NaOH for 2 days at room temperature (20–25°C) in order to remove most of the cellular elements (including epithelial cells) whilst keeping the collagen lamellae intact [9]. The samples were postfixed in 1% osmium tetroxide (O_S_O_4_) for 3 hours and stained by immersion in 0.5% aqueous solution of uranyl acetate for 60 minutes to enhance visualization. The tissue samples were subsequently dehydrated in graded ethanol (ranging from 50% to 100%) and immersed in hexamethyl disilazane (HMDS). Finally, the tissues were coated with a layer of gold in a sputter coater (Polaron system, Quorum Technologies, UK) to enhance structure visualisation. A high vacuum Phillips XL 20 scanning electron microscope (FEI Company, Eindhoven, Netherlands) was used to study the corneal tissue structure at different magnifications.

### Transmission Electron Microscopy and Light Microscopy

Tissue samples taken from the middle of the flap and the residual stromal bed (Figure 1) were immersed in 2.5% glutaraldehyde; after 2 hours, the corneal tissue was removed from the glutaraldehyde and postfixed in 1% osmium tetroxide (O_S_O_4_) followed by 0.5% uranyl acetate. The tissue was then dehydrated in graded ethanol (ranging from 50% to 100%) and embedded in pure Epon resin. Ultrathin sections were cut and stained with uranyl acetate and lead citrate prior to examination in a Phillips EM 208 transmission electron microscope (FEI Company, Eindhoven, Netherlands).

## Results

**Figure 2:**
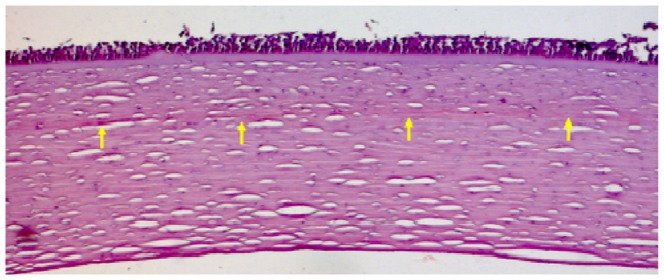
Light microscopy image taken from the central part of a post-mortem LASIK-treated cornea. The flap/stromal bed interface is highlighted by yellow arrows. Original magnification: ×5.

Light microscopy images showed that the flap-bed interface was clearly visible five years after LASIK surgery (Figure 2) and, although some epithelial cells were missing (due to tissue storage and processing), Bowman's layer and Descemet's membrane appeared intact.

**Figure 3:**
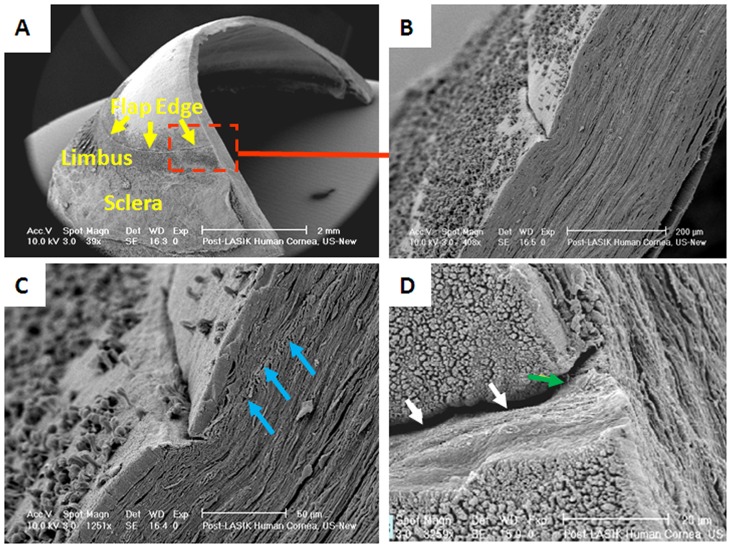
Scanning electron microscopy images of a post-mortem LASIK cornea. The flap edge (yellow arrows in A) can be seen clearly. At higher magnifications, the flap edge appears separated from the adjacent corneal tissue (B). Towards the edge of the flap (at the flap/stromal bed interface), collagen lamellae appear to be in disarray and there is a notable absence of reconnection between adjacent severed lamellae (blue arrows in C). A vertical view of the flap edge shows a gap between the flap margin and the adjacent corneal tissue (white arrows in D). A few bridging fibres are seen to connect the flap to the stromal bed (green arrow in D). The black particles on epithelial and limbal surfaces appear to be adherent basal epithelial cells. Original magnifications for A, B, and C are ×40, ×400, ×1250 and ×3250 respectively.

**Figure 4:**
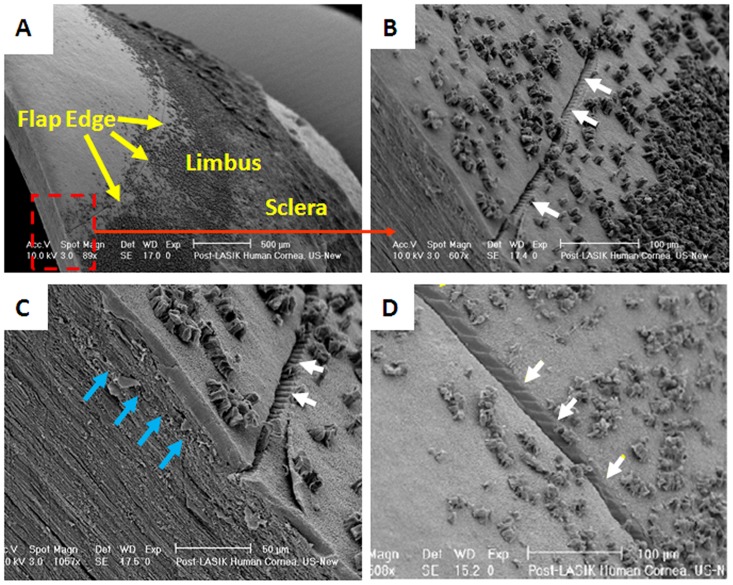
Scanning electron microscopy images showing the primary incision site in a post-mortem LASIK cornea. The flap edge can be easily seen (yellow arrows in A). Examination of the primary incision point (white arrows in B) reveals chatter marks caused by the action of the mechanical microkeratome (white arrows in C and D). Collagen lamellae appear disorganised at the flap/stromal bed interface (blue arrows in C). Original magnifications for A, B, and C are ×90, ×600, ×1050 and ×500 respectively.

When viewed by scanning electron microscopy, the flap was seen to be in close association with the stromal bed, except at the flap margin (Figures 3 and 4), where a tissue gap measuring 10–20 µm in width was observed (Figure 3C–D). Closer examination of the flap and stromal bed interface revealed collagen lamellae to be in disarray with no reconnections formed between adjacent severed lamellae (Figure 3C and 4C). The wound gap at the flap margin showed evidence of a few bridging fibres, but appeared empty of cellular components (Figure 3D). At the primary incision site, chatter lines caused by the action of the mechanical microkeratome were clearly seen in the residual stromal bed (Figure 4D).

**Figure 5:**
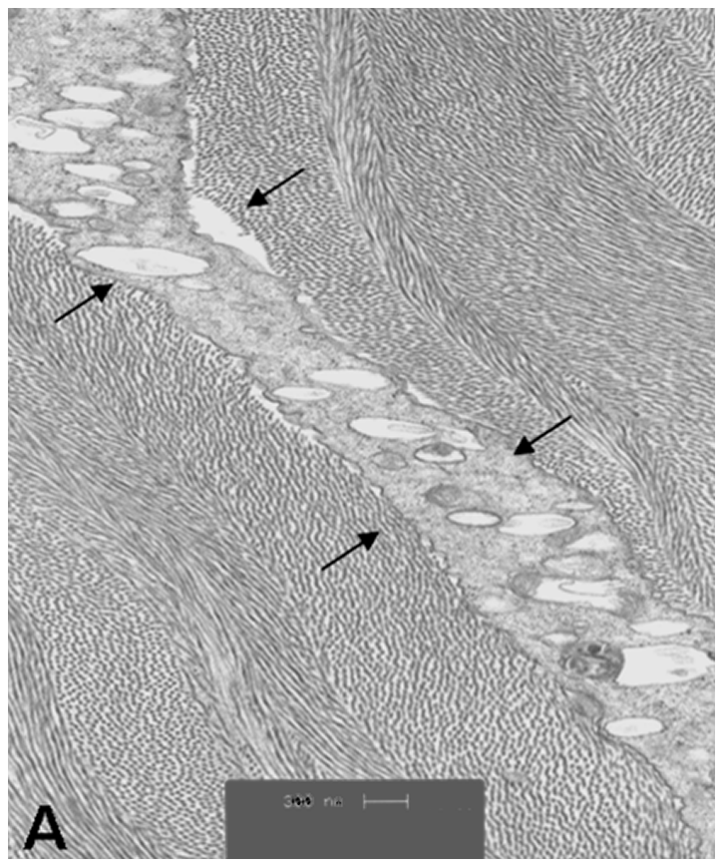
Transmission electron microscopy image of the central wound interface in a post-mortem LASIK cornea. Collagen lamellae lie essentially parallel with the corneal surface on either side of the wound. A primitive scar is formed between the flap and the stromal bed (black arrows). Original magnification is ×16,000.

Transmission electron microscopy images showed stromal collagen lamellae lying essentially parallel to the corneal surface on either side of the wound interface (Figure 5). The interface wound, which measured 1–2 µm thick, appeared free from collagen fibrils or bridging components, but contained primitive scar tissue similar to that reported previously [10] (Figure 5).

## Discussion

Typically, the postoperative results of LASIK surgery are excellent and pain-free with most patients seeing well enough to work or drive without correction the very next day. However, the images presented here show that the LASIK flap can be readily detected 5 years after surgery since only a primitive reparative stromal scar is formed. Unfortunately, due to the limited information supplied to eye banks, most of the clinical details of the patient were unknown, but the results clearly demonstrate the lack of long-term wound healing at the flap margins and across the flap bed. It should however be noted the patient was 50 years old at the time of surgery and there is a significant trend for increased refractive failure with increasing patient age suggesting that stromal changes with age may have influenced post-operative healing [11], [12].

Scanning electron microscopy revealed that the superficial flap edges and the interface wound are visually distinct up to five years after the LASIK procedure and that the flap edges appear empty and separated from the adjunct corneal tissue by a gap of 10–20 µm. This gap was almost certainly formed during scanning electron microscopy processing, as immersion of the tissue in NaOH causes epithelial ingrowth to be removed whilst keeping the collagen lamellae intact. Moreover, it is possible that the tissue fixation process and the subsequent tissue shrinkage may have increased the gap width. It is interesting to note that even after NaOH treatment some cellular material remained at the limbus. The clusters of cells appeared to be basal epithelial cells which are clearly more adherent to the corneal surface than other cells.

Remarkably, even the chatter marks caused by the mechanical microkeratome blade as the flap was created were still visible in the stromal bed (at the primary incision site) once the covering cell layers were removed by NaOH. Previous studies identifying the presence of chatter marks caused by mechanical microkeratomes have shown that the marks are most prominent at the flap margin [13] and become more pronounced after blade reuse [14]. It has been suggested that the tissue irregularities may be related to the development of pressure ridges ahead of the blade [13]. Regardless of the cause, the observed chatter marks provide a remarkable visual demonstration of the lack of connective tissue wound healing at the flap edges following LASIK surgery. In a detailed ultrastructural study of post-mortem LASIK donor corneas, Dawson *et al*. [10] found two types of reparative stromal wound healing responses associated with the LASIK surgery: a hypercellular fibrotic scar at the flap wound margin (which serves as an adhesive to hold the corneal flap in place) and a weaker transparent, hypocellular primitive scar in the remaining paracentral and central areas of the lamellar wound [10]. Our study has shown that collagen lamellae around the wound interface run essentially parallel to the corneal surface. However, the fact that collagen lamellae do not bond with each other at the location of the primitive scar confirms that corneal wound healing after LASIK happens only superficially (in the epithelial cell layers). This helps to explain reports that the biomechanical properties of the post-LASIK cornea are primarily determined by the residual stromal bed and that the LASIK flap contributes only minimally to the overall strength and stability of the cornea [15], [16]. In fact, Schmack et al. [17] showed that on average the central and paracentral hypocellular primitive stromal scar regains only 2.4% of normal stromal strength and displays no evidence of remodelling even 6.5 years after LASIK surgery. The lack of reconnection between severed collagen lamellae at the flap/stromal bed interface also provides an explanation for reported instances of flap dislocations following trauma, which may occur many years after the LASIK surgery [2], [3].

In summary, this study used a technique that removes cellular constituents to highlight the collagenous architecture of the corneal stroma and in doing so identified the ultrastructural features present in human corneas after LASIK surgery. The images presented here clearly show that the LASIK flap does not integrate with the residual stromal bed, even 5 years after surgery.
